# An Assessment of the Relationships Between Umbilical Cord Blood Gas Analysis, APGAR (Appearance, Pulse, Grimace, Activity, and Respiration) Scores, and Neonatal Outcomes

**DOI:** 10.7759/cureus.62362

**Published:** 2024-06-14

**Authors:** Nazan N Doğan Kocabıyık, Ozgul Salihoğlu

**Affiliations:** 1 Department of Pediatrics, Division of Neonatology, University of Health Sciences, Bakırkoy Dr. Sadi Konuk Training and Research Hospital, Istanbul, TUR

**Keywords:** apgar, acidosis, blood gas analysis, umbilical cord blood, newborn

## Abstract

Introduction

Intrapartum hypoxic-ischemic injury is a condition that significantly affects neonatal health and, therefore, needs to be attended to urgently. Umbilical cord blood gas analysis (BGA) results and APGAR (appearance, pulse, grimace, activity, and respiration) scores are commonly used to assess birth asphyxia and the severity of neonatal acidemia. In this context, this study was conducted to investigate the correlations of BGA results and APGAR scores with neonatal outcomes to determine the combined value of BGA results and APGAR scores in neonatal health assessment.

Methods

The sample of this retrospective cohort study consisted of 593 consecutive-term newborns delivered in a tertiary referral center in Turkey between January 2020 and December 2022. All newborns’ maternal, delivery, and neonatal characteristics, BGA results, and APGAR scores were analyzed to determine correlations with composite adverse neonatal outcomes. The study's primary outcome was defined as the rate of the composite adverse neonatal outcomes, whereas the secondary outcomes were determined as the impact of maternal and neonatal characteristics on composite neonatal morbidity and the correlation between the one- and five-minute APGAR scores and umbilical cord BGA parameters.

Results

Of the 593 infants included in the study, 191 (32.2%) infants experienced composite adverse neonatal outcomes, primarily mechanical ventilation (47.7%), followed by respiratory distress/syndrome (35.6%). Significant correlations were detected between composite adverse neonatal outcomes and advanced maternal age (p = 0.025), cesarean section history (p < 0.001), preterm delivery (p < 0.001), lower one- and five-minute APGAR scores (p < 0.001 for both cases), and acidemia severity (p = 0.007). However, the correlations between BGA parameters and APGAR scores were weak (r < 0.2).

Conclusion

This study investigated the correlations between neonatal mortality and morbidity and maternal factors, delivery characteristics, and fetal features, including one- and five-minute APGAR scores and BGA parameters. Nevertheless, weak correlations between BGA parameters and APGAR scores warrant further comprehensive prospective studies.

## Introduction

Intrapartum hypoxic-ischemic injury is one of the leading causes of neonatal morbidity and even neonatal mortality [1,2]. It is vital to assess newborn health immediately after birth. In this context, neonatal umbilical cord blood gas analysis (BGA) has emerged as an essential and definitive diagnostic tool in the evaluation of birth asphyxia and the resulting neonatal acidemia [3-7]. The severity and duration of the hypoxic-ischemic injury are closely associated with the severity of acidemia [8].

BGA parameters differentiating asphyxiated neonates include lactate levels, base deficit, and pH values [2,4,5,9]. Lower pH levels, indicative of neonatal acidemia, have been associated with an increased risk of neonatal morbidity [5,10]. BGA allows differentiating acute and chronic acidemia based on pH values and base deficit [11]. However, there is some controversy about the optimum cut-off values of pH and base deficit that can be used to assess the severity and duration of neonatal acidemia [1,5,7,12,13]. As a matter of fact, a wide range of umbilical pH values from <7.0 to 7.2 were used in the literature to define neonatal acidemia [8,10].

Since its introduction in 1952, the appearance, pulse, grimace, activity, and respiration (APGAR) score has been a fundamental tool for promptly evaluating the physical condition of infants at birth and predicting neonatal survival [14]. APGAR scores assessed one minute and five minutes after the delivery are considered primary indicators for assessing asphyxia, predicting potential neurological damage, and evaluating neonatal vitality [6,12,15]. A five-minute APGAR score below 7 in parallel with supporting BGA results is generally considered to indicate an increased risk of neonatal asphyxia [13,16,17]. However, the findings of the previous studies on the relationship between neonatal acidemia, APGAR scores, and neonatal outcomes are contradictory [4,6,16,17]. The correlations between umbilical BGA results and APGAR scores may not occur under certain conditions, such as acute and chronic intrapartum events or conditions leading to metabolic or respiratory acidosis [7,17-19].

In view of the foregoing, this study was carried out to investigate the correlations of BGA results and APGAR scores with neonatal outcomes in order to determine the combined value of BGA results and APGAR scores in neonatal health assessment.

## Materials and methods

Study design

This study was designed as a retrospective cohort study. The study protocol was approved by the local ethics committee (approval number: 2023-21-25). The study was conducted in accordance with the ethical principles outlined in the World Medical Association’s Declaration of Helsinki. Written informed consent forms could not be obtained from the legal guardians of the infants included in the study due to the study’s retrospective design and the unanimity of data.

Population and sample

The study infants were born at the University of Health Sciences, Dr. Sadi Konuk Training and Research Hospital, Istanbul, Turkey, a tertiary referral center, between January 1, 2020 and December 31, 2022. As an institutional policy, umbilical cord BGA has been routinely performed in all deliveries since 2010. The newborns with complete BGA results, as well as one-minute and five-minute APGAR scores, were included in the sample. Stillbirths, infants with intrauterine growth retardation, significant congenital anomalies, and multiple gestations were excluded from the study [7,8]. We excluded individuals with incomplete data on any variable of the study. In the end, the study sample consisted of 593 term newborns.

Data collection

Neonatal nurses trained in resuscitation were present at all deliveries. Neonatologists attended the deliveries or cesarean section procedures in the event of complicated pregnancies and abnormal neonatal examination findings. The APGAR scoring was performed by Neonatal Resuscitation Program® (NRP®)-certified neonatal nurses and pediatricians.

At least 1.5 ml of umbilical blood were taken into pre-heparinized 2 mL syringes in the labor and delivery room to assess BGA parameters, including the potential of hydrogen (pH), partial pressure of oxygen (PaO_2_) (mmHg), partial pressure of carbon dioxide (PaCO_2_) (mmHg), and bicarbonate (HCO_3_) (mmol/L), base deficit (mmol/L), lactate (mmol/L), and glucose (mg/dL) levels. The cord blood samples were sent to the laboratory immediately after the delivery and analyzed by ABL800 Flex (Radiometer, Copenhagen, Denmark).

The umbilical cord blood samples containing both arterial and venous blood and with an arterial pH value of at least 0.02 less than venous pH values were considered to exclude double sampling of the umbilical artery [7,8].

APGAR scores were assessed one and five minutes after the delivery by either a neonatal nurse attending the delivery or, if present, a neonatologist. An APGAR score cutoff value of 7 was used to categorize the newborns based on the one- and five-minute APGAR scores using the cutoff value of seven [15].

We grouped the newborns based on pH values. Accordingly, newborns with pH ≥7.2 were considered normal and newborns with pH <7.2 were considered to have acidemia [8,10,15]. The severity of acidemia was also categorized based on pH values as severe acidemia (pH ≤ 7.0), moderate acidemia (pH between >7.0 and 7.1), and mild acidemia (pH between 7.1 and <7.2) [8,15]. As for the cutoff values for other BGA parameters, ≥12 mmol/L was used for the base deficit, and ≥5 mmol/L was used for the lactate level [2,5,8,15]. Infants with a birth weight of ≥4000 g were considered to have macrosomia [7].

Maternal characteristics, including demographic information, comorbidities, gestational complications, and obstetric data, i.e., gestational age at delivery and delivery route, were obtained from the medical records via the hospital information system. The neonatal characteristics, such as birth weight, gender, APGAR scores, and neonatal intensive care unit (NICU) stays, were recorded. We also collected data on infant outcomes during the follow-up period up until discharge from the hospital or death. Composite adverse neonatal outcomes included neonatal death, encephalopathy, therapeutic hypothermia, suspected or confirmed sepsis, meconium aspiration syndrome, seizures, invasive respiratory support including mechanical ventilation or continuous positive airway pressure within the first 24 hours following delivery, and respiratory distress [2,7,8].

Neonatal encephalopathy diagnosis was made in accordance with the diagnostic criteria of the American College of Obstetricians and Gynecologists (ACOG) and the American Academy of Pediatrics (AAP) [20]. Respiratory distress diagnosis was made in the event of persistent nasal flaring, subcostal or intercostal retractions, grunting requiring intervention, or need for ventilation support to maintain oxygen saturations of >95% [7]. The diagnosis of meconium aspiration syndrome was made in the event of respiratory distress associated with elevated main pulmonary artery pressures assessed by echocardiography (ECHO) in a meconium-stained amniotic fluid setting before the delivery [8].

The infants included in the sample were grouped based on composite adverse neonatal outcomes. Accordingly, the infants who developed any composite adverse neonatal outcome were included in Group 1 (n = 191), and those without a composite adverse neonatal outcome were included in Group 2 (n = 402).

Statistical analysis

The study’s primary outcome was determined as the rate of the composite adverse neonatal outcomes, whereas the secondary outcomes were defined as the impact of maternal and neonatal characteristics on composite neonatal morbidity and the correlation between the one- and five-minute APGAR scores and umbilical cord BGA parameters. Statistical comparisons were made between neonates with and without an identified composite adverse neonatal outcome in terms of the parameters investigated within the scope of the study. The results were expressed as descriptive statistics. Accordingly, continuous (numerical) variables that did or did not conform to the normal distribution were tabulated using mean ± standard deviation values and median with minimum and maximum values, respectively. In addition, categorical variables were expressed as numbers and percentage values. The normal distribution characteristics of numerical variables were analyzed using Shapiro-Wilk, Kolmogorov-Smirnov, and Anderson-Darling tests.

In order to compare the differences in categorical variables between groups, Pearson’s chi-square test was used in 2 x 2 tables with expected cells of 5 and above, Fisher’s exact test was used in 2 x 2 tables with expected cells less than 5, and the Fisher-Freeman-Halton test was used in RxC tables with expected cells less than 5.

In comparisons between two independent groups, the independent samples t-test was used for normally distributed numerical variables, and the Mann-Whitney U test was used for non-normally distributed numerical variables.

The Spearman correlation coefficients (r) were calculated to analyze the relationships between BGA parameters and the APGAR scores. Accordingly, correlations with r values ≥0.8, from 0.6 to 0.8, 0.3 to 0.5, and <0.3 were considered very strong, strong, moderate, and weak, respectively.

Jamovi project 2.3.28.0 (Jamovi, version 2.3.28.0, 2023, retrieved from https://www.jamovi.org) and JASP 0.17.3 (Jeffreys’ Amazing Statistics Program, version 0.17.3, 2023, retrieved from https://jasp-stats.org) software packages were used in the statistical analyses. Probability (p) statistics of ≤0.05 were deemed to indicate statistical significance.

## Results

Of the 593 infants included in the study, 191 (32.2%) experienced a total of 256 composite adverse neonatal outcomes, predominantly mechanical ventilation (47.7%) and respiratory distress/syndrome (35.6%) (Table 1). Two infants died during the study period, resulting in an overall mortality rate of 0.34%.

**Table 1:** Distribution of the composite adverse neonatal outcomes

Outcomes	Number (n = 256)
Neonatal morbidity, *n(%)*	
Mechanical ventilation	122 (47.7)
Respiratory distress/ respiratory distress syndrome	91 (35.6)
Sepsis	28 (10.9)
Meconium aspiration syndrome	11 (4.3)
Hypoxic ischemic encephalopathy	1 (0.4)
Seizure	1 (0.4)
Neonatal mortality	2 (0.8)

There were significant differences in the demographic and clinical characteristics of infants’ mothers between Groups 1 and 2 (p < 0.05) (Table 2). The mothers of infants in Group 1 were significantly older than those of the infants in Group 2 (p = 0.025). In parallel, the rate of infants whose mothers were over 35 years old was significantly higher in Group 1 than in Group 2 (23.0% vs. 12.7%, p = 0.002). A significant correlation was found between the infants’ mothers having previously given birth by cesarean section and the development of composite adverse neonatal outcomes (p < 0.001). There was no significant difference between the groups in other clinical and obstetric characteristics (p > 0.05). Diabetes and polyhydramnios, which are gestational complications, were significantly more common in the mothers of infants in Group 1 than in those of infants in Group 2 (p = 0.010 and p < 0.001, respectively). There was no significant difference in other gestational complications between Groups 1 and 2 (p > 0.05) (Table 2).

**Table 2:** Maternal demographic and clinical characteristics of the study groups

Characteristics	Groups		p
	Group 1 (n = 191)	Group 2 (n = 402)	
Maternal age (year), *(mean ± SD)*	29.1 ± 6.7	27.8 ± 6.1	0.025
Maternal age <18 year, *n(%)*	3 (1.6)	4 (1.0)	0.686
Maternal age >35 year, *n(%)*	44 (23.0)	51 (12.7)	0.002
Smoking, *n(%)*	21 (11.0)	36 (9.0)	0.523
Cigarette (pack years), m*edian [minimum-maximum]*	5.0 [0.0–25.0]	5.5 [1.0–25.0]	0.969
Comorbidities, *n(%)*			
*Rheumatic*	2 (1.0)	2 (0.5)	0.598
*Thyroid*	10 (5.2)	22 (5.5)	0.999
*Neurological*	7 (3.9)	5 (1.3)	0.061
*Cardiac*	2 (1.0)	2 (0.5)	0.598
*Hematological*	10 (5.2)	9 (2.2)	0.092
Previous cesarean section, *n(%)*	103 (53.9)	123 (30.6)	<0.001
Nulliparity, *n(%)*	35 (18.3)	94 (23.4)	0.198
Gravidity, m*edian [minimum-maximum]*	3.0 [1.0–8.0]	2.0 [1.0–12.0]	0.206
In-vitro fertilization history, *n(%)*	1 (0.5)	2 (0.5)	0.999
Gestational complications. *n(%)*			
Hypertension	12 (6.3)	12 (3.0)	0.093
Diabetes	14 (7.3)	10 (2.5)	0.010
Preeclampsia	9 (4.7)	8 (2.0)	0.112
Cholestasis	2 (1.4)	1 (0.3)	0.245
Placental abruption	1 (0.5)	1 (0.2)	0.541
Oligohydramnios	7 (3.7)	9 (2.2)	0.465
Polyhydramnios	11 (5.8)	2 (0.5)	<0.001

Although there was no significant difference in the median length of the gestational age at delivery (38 weeks) between the groups, the gestational age was significantly shorter in Group 1 than in Group 2 (p < 0.001). Preterm birth was significantly more common among mothers whose infants had adverse neonatal outcomes than among mothers whose infants did not have adverse neonatal outcomes (p < 0.001). The rate of infants whose mothers had a cesarean section was significantly higher in Group 1 than in Group 2 (77.5% vs. 41.5%, p < 0.001). Similarly, the rate of infants whose mothers had an emergency cesarean section was significantly higher in Group 1 than in Group 2 (p < 0.001) (Table 3).

**Table 3:** Obstetric data of the last pregnancy

Variable	Groups		p
	Group 1 (n = 191)	Group 2 (n = 402)	
Gestational age at delivery (week), m*edian [minimum-maximum]*	38.0 [27.0–42.0]	38.0 [35.0–42.0]	<0.001
Gestational age groups, *n(%)*			
<35 weeks	23 (12)	0 (0)	<0.001
≥35 weeks	168 (88)	402 (100)	
Preterm delivery	57 (29.8)	38 (9.5)	<0.001
Mode of delivery,* n(%) *			
Normal vaginal delivery	43 (22.5)	235 (58.5)	<0.001
Cesarean section	148 (77.5)	167 (41.5)	
Emergency cesarean section	31 (16.2)	7 (1.7)	<0.001

There were significant differences in the neonatal characteristics between the groups (Table 4). The rate of male infants was significantly higher in Group 1 than in Group 2 (p = 0.037). The infants in Group 1 had significantly lower birth weights than those in Group 2 (p < 0.001). One- and five-minute APGAR scores were significantly lower in Group 1 than in Group 2 (p < 0.001 for both cases). In parallel, the rates of infants with one- and five-minute APGAR scores below seven were significantly higher in Group 1 than in Group 2 (p < 0.001 for both cases). The rate of infants with an NICU stay was also significantly higher in Group 1 than in Group 2 (p < 0.001).

**Table 4:** Neonatal characteristics of the groups

Characteristics	Groups		p
	Group 1 (n = 191)	Group 2 (n = 402)	
Birth weight (g), *mean ±SD*	3065.5 ± 742.7	3289.6 ± 404.0	<0.001
Macrosomia, *n(%)*	12 (6.9)	16 (4.4)	0.329
Sex,* n(%) *			
*Male*	117 (61.3)	208 (51.7)	0.037
*Female*	74 (38.7)	194 (48.3)	
One-minute APGAR score, m*edian [minimum-maximum]*	7.0 [0.0–9.0]	8.0 [3.0–10.0]	<0.001
Grouping based on the one-minute APGAR score, *n(%)*			
*<7*	54 (28.3)	21 (5.2)	<0.001
*≥7*	137 (71.7)	381 (94.8)	
Five-minute APGAR score, m*edian [minimum-maximum]*	8.0 [2.0–10.0]	9.0 [7.0–10.0]	<0.001
Grouping based on the five-minute APGAR score, *n(%)*			
*<7*	7 (3.7)	0 (0.0)	<0.001
*≥7*	184 (96.3)	402 (100.0)	
Need for NICU, *n(%)*	173 (90.6)	3 (0.7)	<0.001

The infants’ umbilical cord BGA analysis results are shown in Table 5. Accordingly, 26 (4.4%) infants in the overall sample had acidemia. There was no significant difference in the rate of infants with acidemia and umbilical cord blood pH levels between the groups (p = 0.169 and p = 0.362). However, there was no infant with moderate or severe acidemia in Group 2 (p = 0.007). The rate of infants with base deficit was significantly higher in Group 1 than in Group 2 (p = 0.038). There was no significant difference in other BGA parameters between the groups (p > 0.05) (Table 5).

**Table 5:** Comparison of the blood gas analysis findings of the study groups

Cord blood gas components	Groups		p
	Group 1 (n = 191)	Group 2 (n = 402)	
pH, m*edian [minimum-maximum]*	7.3 [7.0–7.5]	7.3 [7.1–7.5]	0.169
pH groups, *n(%)*			
Normal (≥7.2)	180 (94.2)	387 (96.3)	0.362
Acidemia (<7.2)	11 (5.8)	15 (3.7)	
Severity of acidemia, *n(%) *			
Severe (pH ≤ 7.0)	2 (18.2)	0 (0.0)	0.007
Moderate (pH >7.0 to 7.1)	3 (27.3)	0 (0.0)	
Mild (pH >7.1 and <7.2)	6 (54.5)	15 (100.0)	
PaO_2_ (mmHg), m*edian [minimum-maximum]*	29.0 [12.0–120.0]	30.0 [12.0–100.0]	0.746
PaCO_2_ (mmHg), m*edian [minimum-maximum]*	43.0 [26.0–98.0]	43.0 [28.0–75.0]	0.593
HCO_3_ (mmol/L), m*edian [minimum-maximum]*	21.0 [10.8–26.0]	21.2 [4.6–32.0]	0.149
Base deficit (mmol/L), m*edian [minimum-maximum]*	2.9 [0.2–18.2]	2.7 [0.1–19.7]	0.157
Base deficit groups, *n(%)*			
<12 (mmol/L)	186 (97.4)	400 (99.5)	0.038
≥12 (mmol/L)	5 (2.6)	2 (0.5)	
Lactate (mmol/L), m*edian [minimum-maximum]*	2.1 [0.5–14.5]	2.1 [0.6–9.0]	0.796
Lactate groups, *n(%)*			
<5 (mmol/L)	174 (91.1)	378 (94.0)	0.254
≥5 (mmol/L)	17 (8.9)	24 (6.0)	
Glucose (mg/dL), m*edian [minimum-maximum]*	81.0 [39.0–231.0]	82.0 [48.0–186.0]	0.094

There were significant correlations in pH, PaO_2_, PaCO_2_, HCO_3_ values, base deficit, and one- and five-min APGAR scores both within and between the two groups (p < 0.05), yet all were weak, with r values less than 0.2 (Table 6).

**Table 6:** Correlation of the blood gas analysis findings with the one-minute and five-minute APGAR scores

Measurements	Overall (n=593)				Groups							
					Group 1 (n = 191)				Group 2 (n = 402)			
	One-minute APGAR score		Five-minute APGAR score		One-minute APGAR score		Five-minute APGAR score		One-minute APGAR score		Five-minute APGAR score	
	r	p	r	p	r	p	r	p	r	p	r	p
pH	0.181	<0.001	0.172	<0.001	0.205	0.004	0.142	0.049	0.162	0.001	0.171	<0.001
PaO_2_	-0.027	0.509	-0.047	0.250	-0.162	0.025	-0.194	0.007	0.031	0.533	0.014	0.780
PaCO_2_	-0.144	<0.001	-0.142	<0.001	-0.146	0.043	-0.138	0.057	-0.153	0.002	-0.157	0.002
HCO_3_	0.151	<0.001	0.120	0.003	0.163	0.025	0.071	0.331	0.135	0.007	0.120	0.016
Base deficit	-0.145	<0.001	-0.124	0.003	-0.176	0.015	-0.111	0.127	-0.117	0.019	-0.109	0.028
Lactate	-0.019	0.646	-0.031	0.450	-0.100	0.169	-0.040	0.584	-0.006	0.912	-0.052	0.295
Glucose	0.018	0.664	0.019	0.648	-0.005	0.946	-0.024	0.740	-0.019	0.701	-0.010	0.849

## Discussion

The rate of infants with composite adverse neonatal outcomes and mortality rate, which were determined as the study’s primary and one of the secondary outcomes, were found to be 32.2% and 0.34%, respectively. As for the study's other secondary outcomes, several clinical and obstetric characteristics, including advanced maternal age, preterm delivery, a history of cesarean section, giving birth via cesarean section, low one- and five-minute APGAR scores, moderate and severe acidemia, and high base deficit value were found to be significantly associated with composite adverse neonatal outcomes. On the other hand, the study’s last secondary outcome, which was the correlation between the one- and five-minute APGAR scores and umbilical cord BGA parameters, was weak.

Maternal characteristics may predict fetal acidemia and subsequent neonatal morbidity. In the literature, significant correlations were reported between acidemia and maternal characteristics, including advanced maternal age, nulliparity, obesity, diabetes, hypertensive disorders of pregnancy, post-term deliveries, induced labor, placental abruption, and cesarean deliveries [2,8]. In parallel, Zaigham et al. [13] reported a significant positive correlation between five-minute APGAR scores and gestational age. They also reported an inverse correlation between the pH value of umbilical cord blood and gestational age. In comparison, our analysis of the maternal and obstetric characteristics impacting the development of composite neonatal morbidity revealed that older mothers with a history of cesarean section were more likely to have infants with composite adverse neonatal morbidity. Then again, we could not find any significant difference between the mothers of infants with and without composite adverse neonatal morbidity in obstetric history. Our findings suggest that maternal demographic, clinical, and obstetric characteristics may impact fetal acidemia and consequent neonatal morbidity.

There is significant variability between studies in terms of inclusion criteria regarding fetal and birth characteristics. Preterm delivery, one of these characteristics, is known for its prognostic value in predicting fetal health. In fact, many studies investigating the relationship between acidemia and neonatal morbidity have excluded preterm (<37 weeks) births, considering that growth-restricted or preterm fetuses are less tolerant to the hypoxic stress of birth [8,21]. In another study, Bligard et al. [7] investigated the relationship between fetal acidemia and adverse neonatal outcomes in term neonates of mothers who underwent scheduled cesarean delivery. There are also studies evaluating the effects of metabolic acidemia on adverse neonatal outcomes in premature and term babies [2,6]. In comparison, the findings of this study, which included both preterm and term pregnancies, revealed a significant relationship between newborns delivered at a gestational age of less than 35 weeks or preterm birth and composite adverse neonatal morbidity. In this regard, standardized studies adjusted for such confounding variables may yield more clear-cut findings. Since the definition of composite neonatal morbidity varies between studies, some rare conditions considered neonatal morbidity in some studies may not be considered in other studies [2,7,8,21]. In this study, we used the definition provided by Bailey et al. [8], who included several rare conditions, such as grade 3 and 4 intraventricular hemorrhage, periventricular leukomalacia, bronchopulmonary dysplasia, retinopathy of prematurity, and necrotizing enterocolitis among the composite adverse neonatal morbidities. Nevertheless, there was no infant with such rare conditions in our sample. Sepsis and respiratory distress were the most common adverse neonatal morbidities encountered in Bailey et al.’s study group [8], contrary to that of Kraus et al. [2], in which culture-proven sepsis was detected rarely. In comparison, mechanical ventilation, respiratory distress/respiratory distress syndrome, and sepsis were the most frequently observed adverse neonatal morbidities in the infants included in this study. One of the reasons for the differences in the rates of infants diagnosed with acidemia is the differences in the diagnostic criteria for metabolic acidemia [22,23]. We used a simplified pH cutoff value for defining and grading acidemia, as in other studies [7,8,15], for ease of use and simplicity.

The impact of acidemia on composite neonatal morbidity has been investigated in a number of studies [2,5,7,8,10,12,24]. In two of these studies investigating the relationship between mild acidemia and short-term neonatal morbidity in term infants, Bligard et al. [7] and Bailey et al. [8] concluded that acidemia (pH < 7.2) is a significantly poor prognostic indicator with an adjusted relative risk of 2.14 to 2.95 for neonatal morbidity. Similar findings have been reported in the other studies [2]. By contrast, we did not detect a significant relationship between acidemia and composite adverse neonatal outcomes to begin with.

The impact of acidemia severity on neonatal outcomes remains uncertain [8,21]. Although there was no significant difference in pH values between the groups, moderate and severe acidemia were only seen in infants with composite adverse neonatal morbidity. The limited number of infants with moderate and severe acidemia in the study sample prevented meaningful conclusions from being drawn. More comprehensive studies are needed to resolve the uncertainty on this issue.

Lactate or base excess are strong predictors of metabolic acidemia and subsequent neonatal morbidity [8,24,25]. Tuuli et al. [25] demonstrated that lactate level was a better predictor of neonatal morbidity than pH. Many studies have found no significant relationship between pH and base excess <-12 [8]. As a reason, it has been suggested that initial respiratory acidosis in infants with respiratory distress may prevent the development of acidotic metabolic changes. Knutzen et al. [1] reported that incorporating the base deficit parameter into pH parameters for term neonates did not result in additional predictive power in predicting adverse neonatal outcomes. In comparison, we did not find any significant difference in pH or base deficit values between the groups. Then again, the limited number of infants with high base deficit values and moderate and severe acidemia prevented us from reaching a definitive conclusion.

The relationship between APGAR scores and acidemia remains controversial [24]. While a study reported a significant inverse curvilinear relationship between the umbilical cord pH, base excess, and five-minute APGAR score <7 [21], Kraus et al. [2] did not find any significant difference between one- and five-minute APGAR scores in neonates born at ≥35 weeks of gestation irrespective of metabolic acidemia. Similarly, Bligard et al. [7] did not find any significant difference between the APGAR scores of neonates with and without acidemia. A study conducted in Turkey concluded that the well-being of a newborn could not be verified based solely on a five-minute APGAR score of 7 or higher [15]. However, they had included only term neonates without moderate and severe acidemia. Other studies, including this study, have reported very weak or no correlation between BGA parameters and 1- and 5-minute APGAR scores [15,16,26]. The correlation between the one- and five-minute APGAR scores and the umbilical cord pH might be more evident in the event of high-risk pregnancies [6]. In sum, the discrepancies between the studies may be attributed to the wide biological variability in fetal tolerance to hypoxic stress during labor [26].

Limitations of the study

Apart from its strengths, including its relatively large sample size and homogeneous sample due to the exclusion of infants with intrauterine growth retardation and congenital anomalies, there were also some limitations to this study. Its retrospective design and limited number of infants with rare clinical conditions were the study's primary limitations. The fact that long-term outcomes were not investigated may be deemed another limitation.

## Conclusions

This study investigated the correlations between neonatal mortality and morbidity and maternal factors, delivery characteristics, and fetal features, including one- and five-minute APGAR scores and BGA parameters, with an emphasis on the multifactorial nature of fetal acidemia. This research identifies significant correlations between adverse neonatal outcomes and various factors, such as advanced maternal age, cesarean section history, preterm delivery, and lower APGAR scores. Despite revealing important findings, the study underscores the need for more extensive prospective research to enhance the accuracy of neonatal health predictions based on BGA parameters and APGAR scores.

Nevertheless, weak correlations between BGA parameters and APGAR scores warrant further comprehensive prospective studies to refine assessment criteria, enabling more accurate predictions of neonatal morbidity and mortality.
